# New perspectives and future directions in the treatment of heart failure

**DOI:** 10.1007/s10741-019-09829-7

**Published:** 2019-07-20

**Authors:** Pierpaolo Pellicori, Muhammad Javed Iqbal Khan, Fraser John Graham, John G. F. Cleland

**Affiliations:** 1 Robertson Institute of Biostatistics and Clinical Trials Unit, University of Glasgow, University Avenue, Glasgow, G12 8QQ UK; 2 National Heart & Lung Institute and National Institute of Health Research Cardiovascular Biomedical Research Unit, Royal Brompton & Harefield Hospitals, Imperial College, London, UK

**Keywords:** Heart failure, Treatment, Trials, HFpEF, HFrEF

## Abstract

The management of heart failure has changed significantly over the last 30 years, leading to improvements in the quality of life and outcomes, at least for patients with a substantially reduced left ventricular ejection fraction (HFrEF). This has been made possible by the identification of various pathways leading to the development and progression of heart failure, which have been successfully targeted with effective therapies. Meanwhile, many other potential targets of treatment have been identified, and the list is constantly expanding. In this review, we summarise planned and ongoing trials exploring the potential benefit, or harm, of old and new pharmacological interventions that might offer further improvements in treatment for those with HFrEF and extend success to the treatment of patients with heart failure with preserved left ventricular ejection fraction (HFpEF) and other heart failure phenotypes.

**Electronic supplementary material:**

The online version of this article (10.1007/s10741-019-09829-7) contains supplementary material, which is available to authorized users.

## Introduction

Heart failure and its management have changed dramatically over the last 30 years. In the 1980s, patients were included in clinical trials of heart failure based purely on the clinical opinion of the investigator with no objective criteria to confirm the diagnosis. The patients were younger and had fewer comorbidities but a broad range of left ventricular ejection fraction (LVEF) compared with contemporary trials; quality of life was often poor and mortality rate high. Fluid retention, causing peripheral oedema and breathlessness, was the main therapeutic target. Digoxin and diuretics were the only available medical treatments, sometimes accompanied with bed rest and fluid restriction.

Subsequently, objective criteria such as LVEF and, more recently, natriuretic peptides were required to select patients for trials. Initially, trials targeted vasoconstriction, using nitrates and hydralazine [1], and pathologically activated neuro-hormonal systems, using angiotensin-converting enzyme (ACE) inhibitors, angiotensin II receptor blockers (ARBs), beta-blockers and mineralocorticoid antagonists (MRAs). These trials provided evidence that, for heart failure with reduced LVEF (HFrEF), treatment could improve ventricular function, symptoms and signs, as well as morbidity and mortality [2–5]. More recently, other targets and novel treatments have been identified for HFrEF. Ivabradine, an agent that slows the rate of sinus node discharge and therefore heart rate, improved ventricular function, symptoms and morbidity for patients who do not achieve a heart rate < 70 bpm on a beta-blocker; for those with a heart rate > 75 bpm or who were not treated with a beta-blocker, mortality was also reduced [6, 7]. Patients with HFrEF in sinus rhythm with a QRS duration > 130 msec benefitted from cardiac resynchronization therapy (CRT) [8, 9] with improvements in cardiac function, symptoms, morbidity and mortality. Patients who were at low risk of dying for any reason other than an arrhythmia benefitted from an implantable cardioverter-defibrillator (ICD) although its utility is currently being called into question [10, 11]. The development of dedicated specialist HF teams has also been of great importance to inform patients of their diagnosis, prognosis and need for therapy, to improve the implementation of and adherence to treatment and to facilitate titration of medications to target doses, all of which leads to greater patient-satisfaction and better long-term outcomes [12].

Despite these successes, the ‘war’ on heart failure is far from won. For patients hospitalised with worsening heart failure aged less than 75 years, mortality at 1 year may be as high as 20% and up to 40% in those aged > 85 years [13]. For patients with stable HFrEF who survive the initial 6 months after diagnosis and are enrolled in contemporary clinical trials, the annual risk of the composite of hospitalisation for heart failure or mortality is about 10% [14]. Outcome amongst patients who do not participate in clinical trials is much worse [15]. Older patients and those with a recent episode of decompensation despite guideline-recommended therapy who require intensification of therapy have a much worse prognosis. Disappointingly, many patients do not receive, and therefore cannot benefit from, guideline-recommended therapy [16, 17].

More appropriate use of investigations and less complex diagnostic algorithms are likely to reveal that there are many undiagnosed cases of heart failure in the community, particularly with preserved left ventricular (LV) ejection fraction (HFpEF) [18], a condition for which some insist no effective therapy exists as yet, although treatment with a thiazide diuretic and ACE inhibitor exerted remarkable benefits in the HYVET trial in a group of patients many of whom undoubtedly had undeclared HFpEF [19]. Of note, the European Society of Cardiology (ESC) heart failure registry suggested little difference in the therapies applied to patients with HFrEF and HFpEF in clinical practice; perhaps clinicians are sometimes wiser than the guidelines they are asked to follow [20].

The age-adjusted incidence of heart failure may be fairly stable but the total number of patients who will develop heart failure will rise substantially in the next few decades as the proportion of people aged > 60 years increases [21]. Nowadays, many people survive the onset of cardiovascular disease for long periods. Treatment of hypertension, diabetes, chronic kidney disease, atrial fibrillation and ischaemic heart disease might delay the onset of heart failure, but procrastination is not the same as prevention. It is likely that most people with cardiovascular disease will develop heart failure before they die [22, 23]. Strategies to diagnose and treat heart failure before it becomes clinically overt require much more research investment [24]. An increased awareness of what is important to older people may identify novel outcomes and treatments and define the future role of palliative care and euthanasia.

**Fig. 1:**
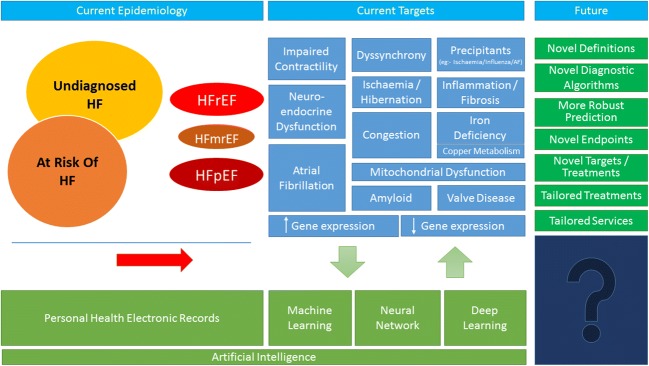
The present and future of heart failure. Conventionally, the prevalence of heart failure is thought to be about 1.5% in the adult population. However, it might be substantially greater than that, as many cases remain undiagnosed, particularly amongst older people, and are usually only identified when symptoms are severe enough to require hospital admission. Several ongoing trials target different pathways that might contribute to disease progression. Success provides tentative insights into the likely mechanisms of progression, although off-target effects may lead to serendipitous effects (this is probably true of most effective treatments for heart failure). There may be many reasons for failure other than the lack of importance of the targeted mechanism. This may include a smaller than anticipated benefit with consequent lack of power, lack of target engagement, a mechanism that is important but only works in a specific subgroup (e.g., heart rate reduction in sinus rhythm) or one that is overwhelmed by competing risks (e.g., rivaroxaban 2.5 mg bd for advanced heart failure in sinus rhythm). Processing large volumes of routinely collected electronic health records using novel analytical approaches, such as artificial intelligence and machine learning, will provide new insights into disease classification, mechanisms of progression and therapeutic targets. Epidemiology, definition and management of heart failure are likely to be transformed in the next decade, with care and services matched to the individual patient’s needs in a “precision-medicine” approach

Enormous amounts of routinely collected personal health records, biochemical and imaging data are now available for novel analytical approaches such as machine-learning and artificial intelligence that will identify novel pathways leading to heart failure and redefine its epidemiology in the next decade (Fig. 1). The definition as well as management of heart failure might be transformed, with care and services personalised to the individual patient’s needs.

Currently, there are many ongoing trials exploring the potential for benefit, or harm, of old and new treatments that might improve the management of HF: summarising novel pharmacological interventions is the purpose of this review; space precludes an in-depth review of devices (electrical, mechanical or valve) or biological interventions (other than influenza vaccination) although key trials are shown in the Table 1 (and in supplementary Table 1, if they aim to enrol fewer than 200 patients).

**Table 1:** Ongoing trials in heart failure (HF). Only trials planned to recruit > 200 participants with HF are shown

Name	ClinicalTrials.gov identifier	Expected completion	Phase	Participants	HF phenotype	Recruitment status
Willingness to participate	NCT03840499	2022	NA	400	All	A
Neuro-endocrine interventions						
Augmentation of natriuretic and other peptides: sacubitril/valsartan						
PARAGON	NCT01920711	2019	3	4822	HFpEF	T
PARALLEL-HF	NCT02468232	2020	3	225	HFrEF	T
PERSPECTIVE	NCT02884206	2022	3	520	HFpEF	A
PARALLAX	NCT03066804	2019	3	2500	HFpEF	A
HFN-LIFE	NCT02816736	2020	4	400	Severe HFrEF	A
Management of hyperkalaemia: patiromer and sodium zirconium cyclosilicate (SCZ)						
DIAMOND (patiromer)	NCT03888066	2022	3	2388	HFrEF	Not yet A
RELIEHF (patiromer)	?	2022/2024	4	400/2000	All	Not yet A
PRIORITIZE HF (SZC)	NCT03532009	Suspended	2	280	HFrEF	A
Vasodilators: vericiguat						
VICTORIA	NCT02861534	2020	3	4872	HFrEF	T
Vitality-HFpEF	NCT03547583	2020	2	735	HFpEF	A
Vasodilators: nitroxyl						
STANDUP-AHF	NCT03016325	2019	2	310	HFrEF	A
Inotropic agents						
Omecamtiv mecarbil						
GALACTIC-HF	NCT02929329	2021	3	8000	HFrEF	A
METEORIC-HF	NCT03759392	2021	3	270	HFrEF	Not yet A
Levosimendan						
LeoDOR	NCT03437226	2019	3	264	HFrEF	A
Digoxin						
DIG-START-AHF	NCT02544815	2019	3	1500	AHF	A
DECISION	NCT03783429	2024	4	982	LVEF < 50%	Not yet A
Recombinant human neuregulin-1β						
	NCT03388593	2023	3	1600	HFrEF	A
Congestion						
Ultrasound guided treatment for congestion						
JECICA	NCT02892227	2019	NA	250	AHF	A
CAVA-ADHF	NCT03140566	2019	NA	388	AHF	A
Device guided treatment for congestion						
GUIDE-HF	NCT03387813	2023	NA	3600	HFrEF and HFpEF	A
Torasemide						
TRANSFORM-HF	NCT03296813	2022	3	6000	HFrEF	A
Acetazolamide						
ADVOR	NCT03505788	2021	4	519	WHF	A
Other combinations of diuretic						
CLOROTIC	NCT01647932	2019	4	304	AHF	A
Spironolactone						
SPIRRIT	NCT02901184	2022	3	3200	HFpEF	A
SPIRIT-HF	2017-000697-11*	?	3	1300	HFmrEF/HFpEF	A
SGLT2i						
Empagliflozin						
EMPERIAL-R	NCT03448419	2019	3	300	HFrEF	A
EMPERIAL-P	NCT03448406	2019	3	300	HFpEF	A
EMMY	NCT03087773	2020	3	476	HF (post AMI)	A
EMPEROR-P	NCT03057951	2021	3	6000	HFpEF	A
EMPEROR-R	NCT03057977	2020	3	2850	HFrEF	A
Sotagliflozin						
SOLOIST-WHF	NCT03521934	2021	3	4000	HFrEF and T2DM	A
Dapagliflozin						
PRESERVED-HF	NCT03030235	2019	4	320	HFpEF	A
DAPA-HF	NCT03036124	2019	3	4744	HFrEF	T
DEFINE-HF	NCT02653482	2019	4	263	HFrEF	T
DELIVER	NCT03619213	2021	3	4700	HFpEF	A
Intravenous iron						
IRONMAN	NCT02642562	2021	3	1300	HFrEF	A
HEART-FID	NCT03037931	2022	3	3014	HFrEF	A
FAIR-HF2	NCT03036462	2020	4	1200	HFrEF	A
FAIR-HFpEF	NCT03074591	2019	2	200	HFpEF	A
Affirm-HF	NCT02937454	2019	4	1100	AHF (LVEF < 50%)	A
Micronutrients: copper, selenium and co-enzyme Q10						
Q10	NCT03133793	2020	2	250	HFpEF	A
TRACER-HF	NCT03875183	2021	2	200	HFrEF	Not yet A
Pulmonary hypertension and right ventricular dysfunction						
Treprostinil						
	NCT03037580	2020	3	310	HFpEF and PHT	A
Macitentan						
SERENADE	NCT03153111	2020	2	300	HFpEF and RV Dysfunction and PHT	A
Cardiac amyloidosis						
Tafamidis-long term	NCT02791230	2024	3	1400	NA	A
Influenza vaccination						
RCT-IVVE	NCT02762851	2020	4	5000	NYHA II-IV	A
INVESTED	NCT02787044	2021	4	9300	HFrEF	A
Hydralazine and metformin						
DANHEART	NCT03514108	2023	4	1500	HFrEF	A
Devices and others						
AdaptResponse	NCT02205359	2023	NA	3700	Adaptive CRT and HFrEF	A
APAF-CRT	NCT02137187	2021	2–3	1830	Atrio-ventricular junction ablation for AF and HF	A
REVIVED-BCIS2	NCT01920048	2022	3	700	IHD and HFrEF (Revasc)	A
GUIDE-CMR	NCT01918215	2023	NA	428	ICD v ILR for HF and LVEF 35–50%	A
RESET-ICD	NCT03494933	2021	NA	2030	CRT-P vs CRT-D	A
RESHAPE-HF2	NCT02444338	2021	NA	420	MR and HFrEF	A
ADVENT-HF	NCT01128816	2020	4	860	Sleep apnoea and LVEF < 45%	A
PURE-HF	NCT03161158	2021	NA	864	HF and severe congestion (venous ultrafiltration)	A

Smaller trials are summarised in Table 1 supplementary

*EUDRACT number

*HFrEF*, heart failure with reduced left ventricular ejection fraction (LVEF); *HFpEF*, heart failure with preserved left ventricular ejection fraction; *HFmrEF*, heart failure with mid-range left ventricular ejection fraction; *AF*, atrial fibrillation; *MR*, mitral regurgitation; *IHD*, ischaemic heart disease; *T2DM*, type 2 diabetes; *ICD*, implantable cardioverter-defibrillator; *ILR*, implantable loop recorder; *CRT*, cardiac resynchronization therapy; *PHT*, pulmonary hypertension; *AHF*, acute heart failure; *AMI*, acute myocardial infarction; *A*, active recruitment; *T*, recruitment terminated

## Neuro-endocrine interventions

### Augmentation of natriuretic and other peptides: sacubitril/valsartan

One of the key therapeutic successes for heart failure has been the inhibition of neuro-endocrine pathways with ACE-Is, ARBs, MRAs and beta-blockers. Recently, a new class of agents, angiotensin receptor neprilysin inhibitors (ARNI), has proved superior to ACE-Is for the treatment of HFrEF [14]. Neprilysin inhibitors retard the degradation of many peptides, including atrial (ANP) and B-type natriuretic peptides (BNP) and vasoactive intestinal polypeptide, which have diuretic, vasodilator and inotropic properties [25, 26]. In the Comparison of Sacubitril–Valsartan versus Enalapril on Effect on NT-proBNP in Patients Stabilized from an Acute Heart Failure Episode (PIONEER-HF) trial, initiation of sacubitril/valsartan for patients with either new-onset or chronic HFrEF (*n* = 881) during the in-hospital recovery phase after an acute decompensation was as safe as initiating enalapril, but led to a greater, and earlier (within 1 week), reduction in plasma concentrations of NT-proBNP, which was sustained until the end of 8 weeks follow-up [27]. A reduction in a composite of serious HF-related adverse clinical events was also observed [28]. However, about 20% of surviving patients discontinued treatment with either ACEi or ARNI and only 55% achieved guideline-recommended doses of the ARNI [27]. In the PRIME trial (*n* = 118), patients with HF, an LVEF < 50% and functional mitral regurgitation (MR) who were randomised to sacubitril/valsartan had a greater reduction in the effective regurgitant orifice area (EROA) compared with valsartan alone at 12 months follow-up [29]. Other trials are currently ongoing in specific populations with HFrEF, including those with symptoms at rest (NCT02816736), or an elevated pulmonary artery pressure (NCT02788656) or in Japan (NCT02468232).

The Prospective Comparison of ARNI with ARB Global Outcomes in HF With Preserved Ejection Fraction (PARAGON; NCT01920711) is a randomised, double-blind, event-driven trial comparing the efficacy and safety of valsartan vs sacubitril/valsartan in patients with HFpEF that has enrolled 4822 patients (mean age 73 ± 8 years, median NT-proBNP 911 (interquartile range 464–1610) pg/mL, > 2/3 in sinus rhythm) [30]. The results should be reported later in 2019. PARALLAX (NCT03066804) is another large (> 2,000 patients) randomised, double-blind trial of patients with HFpEF, comparing sacubitril/valsartan with a control group (the investigator can chose whether this is an ACE-I, an ARB or neither, in which case patients assigned to the control group receive placebo); the effect on plasma NT-proBNP and exercise capacity after 24 weeks of treatment and safety are the main outcomes of interest.

Concerns exist that the inhibition of *neprilysin* could interfere with breakdown of beta amyloid (βA) peptides, which might accumulate in the brain and contribute to the development of Alzheimer’s disease. The PERSPECTIVE trial (NCT02884206) is currently recruiting ~ 500 patients with HF and LVEF > 40%, to investigate whether chronic administration of sacubitril/valsartan for 3 years leads to a decline in cognitive function when compared with valsartan alone.

### Management of hyperkalaemia: patiromer and sodium zirconium cyclosilicate

Currently, based on the evidence provided by clinical trials, guidelines recommend that ACEi, ARB and MRA should not be initiated if serum potassium is > 5.0 mmol/L (5.2 mmol/L for ARNI) and that doses should be reduced or treatment stopped if serum potassium is > 5.5 mmol/L. Accordingly, many patients with HFrEF do not receive guideline-recommended doses of these agents [16, 17, 31]. Older patients, those with type-2 diabetes mellitus and those with renal dysfunction are more likely to develop hyperkalaemia [32]. Patients who fail to achieve guideline-recommended doses of these medications due to hyperkalaemia have a worse prognosis, but this may be because of concomitant renal dysfunction or hypotension.

Patiromer and sodium zirconium cyclosilicate are novel oral treatments that bind potassium in the gastrointestinal (GI) tract and rapidly normalise serum potassium concentrations. Whether their use will allow doctors to prescribe and patients to achieve guideline-recommended doses of RAASi more often and whether this will improve outcomes are now being investigated. Results of substantial trials are not expected before 2021.

### Vasodilators: vericiguat and nitroxyl

Nitric oxide (NO) activates soluble guanylate cyclase (sGC), causing an elevation of intracellular cyclic guanosine monophosphate (cGMP) in vascular and non-vascular tissues, such as the myocardium and kidney. In heart failure, production of NO is reduced and its degradation is increased, leading to an increase in systemic and pulmonary arteriolar and venous tone, thereby increasing the after-load and pre-load on the failing myocardium [33]. Vericiguat is an oral sGC stimulator which increases cGMP production. Phase 2 trials showed that vericiguat is well tolerated in patients with HFrEF [34]. A large (~ 4,500 patients) phase 3 trial (VICTORIA; NCT02861534) is currently evaluating whether vericiguat improves morbidity and mortality compared with placebo in patients with chronic HFrEF [35].

Nitroxyl is a second-generation donor of nitric oxide that causes vasodilatation and may have inotropic effects, which are only partially mediated by an increase in cGMP [36]. A phase 2 trial (STAND-UP; NCT03016325) is currently evaluating the safety and efficacy (changes in NT-proBNP and symptoms) of 48-h infusion of nitroxyl in 310 patients admitted with decompensated HFrEF. Smaller mechanistic trials are investigating its effects on cardiac and renal function.

## Inotropic agents

### Omecamtiv mecarbil, levosimendan, digoxin and recombinant human neuregulin-1

Omecamtiv mecarbil (OM) is a cardiac myosin activator that alters the kinetics of actin/myosin cross-bridges, prolonging the duration of the systole and, thus, stroke volume, without increasing ATP consumption [37]. Phase II trials showed that IV administration of OM in patients with acutely decompensated HFrEF had the expected haemodynamic effects but no clear clinical benefit [38]. In The Chronic Oral Study of Myosin Activation to Increase Contractility in Heart Failure (COSMIC-HF) trial, oral OM given for 20 weeks was safe and reduced LV size and plasma concentrations of NT-proBNP levels; the latter effect persisted for 4 weeks after treatment withdrawal suggesting that long-term favourable structural remodelling had occurred [39]. The Phase II trial programme has repeatedly shown small increases in serum troponin concentrations, raising concerns about safety that, so far, appears unfounded. Increases in troponin appear unrelated to any clinical evidence of myocardial ischaemia or adverse outcomes. A large (*n* ~ 8,000) phase III trial of patients with chronic HFrEF (with 25% planned to be enrolled during a hospitalisation for an episode of decompensation) is nearing completion of enrolment and should report in 2021 (GALACTIC-HF; NCT02929329).

Levosimendan, a vasodilator and calcium sensitiser, has been used to treat refractory HF in many countries despite two large neutral trials conducted in patients with acute HF and a large trial of an oral formulation in patients with chronic severe HF that showed reductions in NT-proBNP and an improvement in QoL but did not otherwise improve outcome [40, 41]. Recently, small trials have explored the effects of giving levosimendan intermittently to patients with chronic severe HFrEF and shown that this can reduce plasma concentrations of NT-proBNP [42]. Larger trials are now attempting to determine whether this strategy can improve symptoms, exercise capacity, morbidity and mortality in patients with HFrEF.

Neuregulin-1 proteins are important for the development and function of cardiac myocytes. Small phase II studies reported that recombinant human neuregulin-1 improved haemodynamics and promoted reverse LV remodelling in patients with HFrEF [43, 44]. A phase III study is currently testing whether, compared to placebo, use of daily (for 10 days) IV infusions, followed by weekly boluses, of recombinant human neuregulin-1 is feasible, safe and effective in reducing mortality in Chinese patients with mild to moderate chronic HFrEF.

Digoxin may be the oldest medicine still prescribed for heart failure, but controversies persist about its benefits. In the DIG trial, conducted before many current HF treatments were available, digoxin did not reduce mortality compared to placebo, although it did reduce HF hospitalisations by 28%. A retrospective analysis suggested that patients with serum concentrations of digoxin of 0.5–0.9 ng/mL were more likely to benefit [45, 46]. A prospective, randomised, placebo-controlled trial is testing whether lower doses of digoxin, guided by measurements of its plasma concentrations (0.5–0.9 ng/mL), will reduce HF hospitalisations and cardiovascular death in ~ 1,000 symptomatic patients with chronic HF and a reduced or mid-range LVEF (< 50%) (NCT03783429).

## Congestion

Congestion is an important cause of the symptoms and signs of HF, leads to adverse atrial and ventricular remodelling, arrhythmias and worsening renal function and is associated with poor outcomes [47, 48]. Controlling congestion is a key therapeutic goal in the management of heart failure. However, clinical identification of congestion is challenging, unless severe. Up to 50% of outpatients with HF who were considered to be clinically dry had sub-clinical congestion on ultrasound, either in the pulmonary interstitium (lung B-lines) or in the intra-vascular space, as measured by a distended inferior vena cava (IVC). Sub-clinical congestion was associated with a poor outcome [49, 50]. Whether treatment guided by ultrasound assessments is feasible and effective for the management of congestion in patients with HF is currently being explored in several small- to medium-sized trials. Biomarker-guided management of congestion has met with mixed success, largely because treatment was similarly effective in each arm [51]. A large trial (GUIDE-HF; NCT03387813) is currently investigating whether pulmonary artery pressure monitoring using a small implanted device can help guide treatment of congestion.

### Torasemide, acetazolamide and other diuretics

Loop diuretics are the most potent diuretic agents, and furosemide is the most widely used in patients with HF. However, other loop diuretics, such as bumetanide and torasemide, are either better absorbed or delivered more reliably to the renal tubule. Meta-analysis of small randomised trials and observational studies suggests that torasemide might be superior to furosemide, but no substantial randomised trial has yet compared these two agents [52–54]. TRANSFORM-HF (NCT03296813) is an ongoing, multi-centre, unblinded, trial that will randomise, prior to discharge, ~ 6000 patients admitted with decompensated heart failure to long-term treatment with oral torasemide or furosemide to investigate effects on morbidity and mortality.

Other options for treating resistant congestion in patients HF exist, such as combining different classes of diuretics, but their safety and efficacy have been rarely tested in clinical trials [55]. Most of the sodium filtered by kidneys is reabsorbed in the proximal tubule of the nephron. Acetazolamide, a carbonic anhydrase inhibitor, should decrease the amount of sodium reabsorbed in the proximal nephron and enhance the distal effects of loop diuretics. The Acetazolamide in Decompensated heart failure with Volume OveRload (ADVOR) is a randomised, double-blind, placebo-controlled trial which will test whether combining acetazolamide with a loop diuretic is more successful in achieving decongestion in ~ 500 patients admitted with HF and signs of fluid overload [56].

### Sodium glucose co-transporter 2 inhibitors

Although not everyone would agree that it is the principal mechanism of action of sodium glucose co-transporter 2 inhibitors (SGLT2i), there is little doubt that diuresis contributes to their effects in HF. SGLT2i reduce glucose reabsorption in the proximal nephron, increasing delivery of glucose and sodium to the distal nephron and inducing an osmotic diuresis. Whether SGLT2i have additional metabolic effects on the heart and kidney by inhibiting carbonic anhydrase or increasing the availability of ketones as a metabolic substrate for the myocardium is uncertain [57]. Empagliflozin reduced all-cause mortality and hospitalisation for heart failure in patients with type 2 diabetes mellitus (T2DM) and ischaemic heart disease (IHD) [58]. Trials of canagliflozin and dapagliflozin also suggested a reduction in hospitalisations for HF [59–61]; although the relative risk reduction was substantial, the absolute benefits were very small, creating uncertainty about whether they are clinically meaningful. Interestingly, the programme of phase III trials for HF has not required patients to have T2DM and has enrolled a broad range of patients with HFrEF and HFpEF as well as in-patients and out-patients. The first of these trials is likely to report in 2019 (DAPA-HF) [62].

## Intravenous iron

Up to 50% of patients with HF have iron deficiency (ID), with or without anaemia. ID is associated with adverse outcomes, even in the absence of anaemia, and is a potential target of treatment [63]. Oral iron is widely available and cheap but only a small amount of oral iron can be absorbed in a day (perhaps 2–10 mg/day compared with a total deficiency of > 1,000 mg) and many patients have GI intolerance to oral iron. Oral iron absorption may be impaired in heart failure, possibly due to increased secretion of hepatic hepcidin, but even if it is not, oral supplementation would take many months to correct iron deficiency [64]. Modern preparations of IV iron are safe and well tolerated and improve symptoms and exercise capacity in patients with HFrEF. An individual patient meta-analysis from four randomised controlled trials including 839 patients with HFrEF and ID, of whom 504 were randomised to IV ferric carboxymaltose, suggests that short-term (mean follow-up 31 weeks) treatment could also reduce HF hospitalisations when compared with placebo. However, the analysis included very few cardiovascular (*n* = 34) or other (*n* = 4) deaths and does not prove long-term safety [65]. Four substantial (> 1000 patients) randomised trials are currently investigating whether different formulations of IV iron (either iron isomaltoside or ferric carboxymaltose) improve morbidity and mortality in patients with chronic or acute HF. These trials have included far more patients and recorded far more events than the published evidence but have not yet been stopped for benefit. Phase II trials are also investigating the potential benefits of IV iron on symptoms, exercise tolerance and quality of life of patients with HFpEF and ID (NCT03074591).

## Copper, selenium and co-enzyme Q10

Heart failure may be accompanied by high plasma copper concentrations but myocardial copper depletion. There is evidence from both animal models and a limited amount of human data that copper chelation may be beneficial [66]. However, an alternative view is that low doses of the chelating agent trientine might facilitate copper redistribution to tissues. This concept is currently being tested in a 200-patient, dose-ranging trial (NCT03875183).

Co-enzyme Q10 is an essential component of the mitochondrial electron transport chain and both co-enzyme Q10 and selenium have an important role in many metabolic processes. Lower plasma concentrations of Q10 and selenium have been associated with adverse outcomes in heart failure [67–69]. Two trials showed a reduction in mortality with co-enzyme Q10 supplements for patients with or at high-risk of heart failure and a broad range of LVEF [70, 71]. Randomised controlled trials are underway.

## Other trials

### Pulmonary hypertension and right ventricular dysfunction

Pulmonary hypertension (PHT) is common, especially in patients with advanced heart failure, due to a combination of left atrial hypertension, pulmonary arteriolar hypertrophy and pulmonary vasoconstriction. Small trials have shown that sildenafil, a selective inhibitor of type 5 phosphodiesterase, might improve haemodynamics and exercise performance in patients with HFrEF and PHT; other trials should report soon [72]. In HFpEF, sildenafil was not beneficial [73]. The effects of treprostinil, a synthetic analogue of prostacyclin with potent vasodilator properties, on exercise capacity and NT-proBNP are currently under investigation in a trial (*n* ~ 300) of HFpEF and PHT. However, trials in patients with HFrEF were stopped for harm. The safety, and effect on NT-proBNP levels of macitentan, an antagonist/blocker of endothelin receptors, will be also studied in 300 patients with HFpEF complicated by PHT or right ventricular dysfunction (SERENADE, NCT03153111).

### Amyloidosis

Accumulation of wild-type or variant transthyretin amyloid occurs when fibrils become unstable and misfold. Recent reports suggest that 15–20% of patients with HFpEF may have TTR amyloidosis. These patients have a poor outcome and may not respond to conventional treatments [74]. A recent trial showed that treatment with tafamidis, which binds to transthyretin, preventing tetramer dissociation and amyloidogenesis, improves symptoms, quality of life and exercise capacity and reduces cardiovascular hospitalisations and mortality in patients with transthyretin amyloid cardiomyopathy [75]. The costs of tafamidis are currently prohibitive, preventing large-scale uptake. However, demonstration of the effectiveness of treatment will lead to changes in diagnostic pathways (at least to identify patients who may not benefit from some treatments or for selection into clinical trial even if treatment is unaffordable). In due course, the cost of tafamidis will fall.

### Influenza vaccination

Influenza might be an important precipitant of HF hospitalisations [76]. A recent observational study from Denmark suggested that influenza vaccination might be associated with better outcomes in patients with heart failure, but it also reported that a large proportion (> 40%) of patients with heart failure do not receive influenza vaccination, which might reflect lack of evidence arising from trials and therefore weak recommendations from guidelines [77]. Two large trials investigating the ability of influenza vaccinations to reduce morbidity and mortality should report in the next few years. The Influenza Vaccine To Prevent Adverse Vascular Events (RCT-IVVE) will randomise ~ 5,000 patients with HF globally. The INfluenza Vaccine to Effectively Stop Cardio Thoracic Events and Decompensated Heart Failure (INVESTED) will compare high-dose trivalent influenza vaccine vs standard-dose quadrivalent influenza vaccine in almost 10,000 patients with a recent myocardial infarction or hospitalisation for HF.

## Conclusions

Over the last 30 years, various pathways leading to the development and progression of heart failure have been identified and successfully targeted with effective therapies. This has improved the quality of life and survival for millions of individuals with HFrEF, globally. Hopefully, new treatments will offer further improvements and extend these successes to the treatment of HFpEF and other specific causes and phenotypes of HF. New concepts of how HF should be defined combined with new analytical approaches using large data-sets will re-shape its epidemiology and offer new therapeutic targets. However, old age rather than cardiac dysfunction may be the next great barrier to overcome.

## Electronic supplementary material

ESM 1 (DOCX 23 kb)
