# Supplementary material for: New perspectives and future directions in the treatment of heart failure
Source: Heart Fail Rev. 2019 Jul 20;25(1):147–59. doi: 10.1007/s10741-019-09829-7 (PMC6985084; doi:10.1007/s10741-019-09829-7)
Supplement: Supplementary file 1 — (DOCX 23 kb) [file 10741_2019_9829_MOESM1_ESM.docx]

| Name | ClinicalTrials.gov Identifier: | Expected  completion | Phase | Participants | HF phenotype | Recruitment Status |  |
| --- | --- | --- | --- | --- | --- | --- | --- |
| **Neuro-Endocrine Interventions** | | | | | | | |
| **Augmentation of natriuretic and other peptides: Sacubitril/Valsartan** | | | | | | | |
| PARENT | NCT02788656 | 2019 | NA(Pilot) | 20 | HFrEF & PHT | A |  |
| **Vasodilators: *Nitroxyl*** | | | | | | | |
|  | NCT03730961 | 2019 | 2 | 20 | HFrEF & CKD | A |  |
|  | NCT03357731 | 2019 | 2 | 42 | HFrEF | T |  |
| Inotropic Agents | | | | | | | |
| **Levosimendan** | | | | | | | |
| LOCO-CHF | NCT03576677 | 2020 | 4 | 42 | HFrEF | A |  |
| HELP | NCT03541603 | 2019 | 2 | 36 | HFpEF & PHT | A |  |
| Korea Bridging Study | NCT03555123 | 2019 | 3 | 112 | AHF | Not Yet A |  |
| **Congestion** | | | | | | | |
| **Ultrasound guided treatment for congestion** | | | | | | | |
| CLUSTER-HF | NCT03613779 | 2019 | NA | 182 | AHF | A |  |
| BLUSHED-AHF | NCT03136198 | 2019 | 2 | 130 | AHF | T |  |
| N-FURIOUS | NCT03259165 | 2019 | 2 | 70 | AHF | A |  |
| **Acetazolamide** | | | | | | | |
| ACETA | NCT03720288 | 2020 | 3 | 90 | HFrEF, WHF | A |  |
| **Other combination of diuretic** | | | | | | | |
| FUROSEMIDE SC | NCT03359161 | 2019 | 2-3 | 60 | All | A |  |
| LD+METOLAZONE | NCT03746002 | 2019 | 4 | 60 | WHF | A |  |
| **SGLT2i** | | | | | | | |
| **Empagliflozin** | | | | | | | |
| EMBRACE-HF | NCT03030222 | 2019 | 4 | 60 | HF & CardioMEM | A |  |
|  | NCT03753087 | 2020 | 4 | 100 | HFpEF & T2DM | A |  |
| EMPA-RESPONSE | NCT03200860 | 2020 | 2 | 80 | AHF/WHF | A |  |
| EMPIRE-HF | NCT03198585 | 2019 | 2 | 189 | HFrEF | A |  |
| ERA-HF | NCT03271879 | 2020 | 4 | 128 | HFrEF | Not yet A |  |
| **Sotagliflozin** | | | | | | | |
|  | NCT03292653 | 2019 | 2 | 81 | WHF | A |  |
| **Intravenous/Oral Iron** | | | | | | | |
| PREFER-HF (IV) | NCT03833336 | 2020 | 3 | 72 | HFpEF | A |  |
| ORION-LVAD-1 (oral) | NCT03774615 | 2020 | 4 | 25 | Severe HF (LVAD) | A |  |
| IronEx (IV) | NCT03803111 | 2020 | NA | 64 | HFrEF | Not Yet A |  |
| IRON-CRT (IV) | NCT03380520 | 2019 | 4 | 100 | HFrEF & CRT | A |  |
| **Pulmonary hypertension and right ventricular dysfunction** | | | | | | | |
| **Sildenafil** | | | | | | | |
| SIL-HF | NCT01616381 | 2019 | 3 | 75 | HFrEF & PHT | T |  |
| SIL-HF-US | NCT03460470 | 2019 | 3 | 25 | HFrEF & PHT | A |  |
| REVAD | NCT03356353 | 2019 | 3 | 24 | HFrEF & LVAD | A |  |
|  | NCT01913847 | 2019 | 3 | 144 | HFrEF & PHT | A |  |
| **Cardiac Amyloidosis** | | | | | | | |
| Doxycycline and Tauroursodeoxycholic Acid | NCT03481972 | 2021 | 3 | 102 | NA | A |  |
| Doxycycline | NCT03474458 | 2021 | 2-3 | 120 | NA | A |  |
| Inotersen | NCT03702829 | 2021 | 2 | 50 | NA | Not Yet A |  |
| **Myeloperoxidase inhibitor** | | | | | | | |
| SATELLITE | NCT03756285 | 2020 | 2 | 96 | HFpEF | A |  |
| **Gut Microbiota** | | | | | | | |
| GutHeart (Rifamicin) | NCT02637167 | 2019 | 2 | 150 | HFrEF | A |  |

**Supplementary Table 1. Ongoing trials in heart failure (HF).** Only trials planned to recruit < 200 participants with HF are shown. Larger trials are summarised in Table 1. Abbreviations used: Heart Failure with Reduced Left Ventricular Ejection Fraction (LVEF) – HFrEF; Heart Failure with Preserved Left Ventricular Ejection Fraction – HFpEF; Chronic kidney disease*-* CKD*;* Type 2 diabetes – T2DM; cardiac resynchronization therapy - CRT; Pulmonary Hypertension – PHT; Acute Heart Failure – AHF; Worsening Heart Failure – WHF; Left ventricular assist device*-* LVAD; Active Recruitment – A; Recruitment terminated – T.
